# Multi-dose enteral L-citrulline administration in premature infants at risk of developing pulmonary hypertension associated with bronchopulmonary dysplasia

**DOI:** 10.21203/rs.3.rs-3006963/v1

**Published:** 2023-06-09

**Authors:** Judy Aschner, Charul Avachat, Angela Birnbaum, Catherine Sherwin, Candice Fike

**Affiliations:** University of Minnesota; University of Minnesota; Univ Utah

**Keywords:** pharmacokinetics, nitric oxide, L-arginine

## Abstract

**Objective.:**

Information is needed to guide the design of randomized controlled trials (RCTs) evaluating L-citrulline as a therapy for premature infants with pulmonary hypertension associated with bronchopulmonary dysplasia (BPD-PH). Our goal was to evaluate the tolerability and ability to achieve a target steady-state L-citrulline plasma concentration in prematures treated enterally with a multi-dose L-citrulline strategy based on our single-dose pharmacokinetic study.

**Study Design.:**

Six prematures received 60 mg/kg of L-citrulline every 6 hours for 72 hours. Plasma L-citrulline concentrations were measured before the first and last L-citrulline doses. L-citrulline concentrations were compared to concentration-time profiles from our previous study.

**Results.:**

Plasma L-citrulline concentrations agreed with the simulated concentration-time profiles. No serious adverse events occurred.

**Conclusions.:**

Simulations based on single-doses can be used to predict target multi-dose plasma L-citrulline concentrations. These results assist the design of RCTs evaluating the safety and effectiveness of L-citrulline therapy for BPD-PH. Clinical trials.gov ID: NCT03542812

## INTRODUCTION

Premature infants with chronic lung disease of prematurity, bronchopulmonary dysplasia (BPD), are at risk of developing pulmonary hypertension (PH)^1, 2, 3, 4, 5, 6^. It has been estimated that between 8–42% of infants with BPD have evidence of PH^1, 2, 3, 4, 6^. Mortality rates for infants with BPD-PH have been reported to be as high as 47%^3, 4, 6, 7, 8^. In addition, there is a growing appreciation that the individual and societal costs associated with BPD-PH are substantial^9, 10^.

Research efforts are needed to develop and evaluate therapies to improve outcomes for infants with BPD-PH. In 2022, the Pediatric Pulmonary Hypertension Network (PPHNet) noted the remarkable contribution of BPD-PH to the spectrum of pediatric PH and highlighted the need for focused research and randomized clinical trials (RCTs) in this understudied group of patients^11^. To date, no RCTs have been performed to evaluate the effi cacy of any PH-targeted pharmacotherapy as a treatment to inhibit or reverse the development of BPD-PH^12^. Per FDA guidance, prior to performing a RCT, pharmacokinetic studies are needed to establish doses to be evaluated for effi cacy and safety of any drug in a pediatric population^13^.

Nitric oxide (NO) is a potent pulmonary vasodilator that can be administered exogenously as inhaled NO (iNO) gas. Oral/enteral supplementation with the NO-L-arginine precursor, L-citrulline, provides a way to increase endogenous NO as a potential treatment for PH^14^. Proof of concept to evaluate enterally administered L-citrulline as a PH-targeted therapy is provided by studies with a newborn piglet model showing that oral L-citrulline increased pulmonary vascular NO production and inhibited both the onset and progressive development of chronic hypoxia-induced PH^15, 16^.

Our overarching goal is to evaluate orally/enterally administered L-citrulline as a potential treatment for BPD-PH. As a first step, we recently published data from a phase I study that characterized the PK profile of a single dose of enterally administered L-citrulline in premature infants at risk of developing BPD-PH to derive optimal dosage strategies using simulation-based methodology^17^. The current study aimed to characterize the tolerability and ability to achieve target steady-state trough L-citrulline plasma concentrations when premature infants at risk of developing BPD-PH were treated for 72 hours with multiple doses of enteral L-citrulline using a dosage strategy based on simulations from our previous pharmacokinetics study^17^. This information is needed in order to better inform the design of future RCTs that will evaluate safety and effi cacy.

## Materials/Subjects and Methods

### Patient Population.

This study was the multiple-dose steady state arm of a phase I study NCT03542812 (PK of L-citrulline in infants at substantial risk of developing PH associated with BPD). The single-dose PK arm of the study has been previously published^17^.

We enrolled participants from two Newborn Intensive Care Units (NICUs) in Salt Lake City, UT, the University of Utah Hospital NICU and the Intermountain Medical Center NICU. The institutional review board for human subjects committee at each hospital approved the study protocol. Informed consent was obtained from the legal guardians of all subjects.

Premature neonates born at ≤ 28 weeks gestation were eligible to be enrolled in the study if they required invasive mechanical ventilation or non-invasive positive pressure support (nasal continuous positive airway pressure or high flow nasal cannula ≥ 1 liter per minute) at 32 ± 1 week’s postmenstrual age. Other criteria included (1) the ability to tolerate at least one-half of full-volume oral/gavage tube feedings, using 120 ml/kg/d as full-volume oral/gavage tube feedings, (2) a need for some form of continuous respiratory support for the prior 14 days, and (3) a hemoglobin ≥ 10 mg/dl.

Multiple births and infants with anticipated death prior to hospital discharge, were excluded from the study. Also excluded were patients with any known major fetal anomalies, chromosomal aneuploidy, or clinical evidence of congenital heart disease, except for patent ductus arteriosus, atrial septal defect, or ventricular septal defect. Additional exclusion criteria were the presence of any acute illness as defined by a fever > 100.4°, vomiting or diarrhea, a urine output < 1 ml/kg/hr., significant feeding intolerance beyond the first week of life, or a history of or known to have liver failure or necrotizing enterocolitis.

### Patient monitoring.

Vital signs (heart rate, respiratory rate, temperature, blood pressure, and oxygen saturation) and respiratory support (FiO_2_, mode of support e.g.ventilator, continuous positive airway pressure (CPAP), or high flow nasal cannula (HFNC); and specific settings e.g. cm H_2_O for CPAP or flow for HFNC) were recorded every 6 hours during the 72 hours of study drug administration and every 12 hours for 24 hours post study drug administration.

L-citrulline administration carries a theoretical risk of systemic hypotension. An adverse drop in blood pressure was defined as a decrease in mean arterial blood pressure of more than 25% of baseline blood pressure. Enterally administered medications carry the risk of causing gastro-intestinal disturbances, such as vomiting and diarrhea. Therefore, participants were monitored for clinical evidence of feeding intolerance (including vomiting and diarrhea), necrotizing enterocolitis, and gastrointestinal bleeding. Participants were also monitored for clinical evidence of sepsis. Adverse events were recorded during treatment and within 48 hours at the end of treatment. The Principal Investigator determined any potential relationship to L-citrulline treatment. In addition, a data safety monitoring committee reviewed each adverse event and its relationship to L-citrulline. An interim safety analysis was undertaken after the first 3 participants were enrolled and after a total of 6 participants were enrolled.

### Drug dosing and sample collection.

Each patient received 12 doses of 60 mg/kg enteral L-citrulline. The L-citrulline was donated by Askeplion Pharmaceuticals, Baltimore, MD, and was provided in a powder form that was solubilized in sterile water to achieve 50 mg/ml concentration. Each participant received 1.2 ml/kg (60 mg/kg) of the solubilized L-citrulline every 6 hours ± 30 min (4 times a day) for 72 hours. The total daily volume of solubilized L-citrulline was 4.8 ml/kg/d for a total daily L-citrulline dose of 240 mg/kg/d. Each dose of L-citrulline was delivered via an indwelling nasogastric feeding tube thirty minutes prior to a feeding.

Two blood samples were collected into EDTA microtainers by heel stick methodology from each infant. The first sample was a pre-dose blood sample collected between 10 minutes and 6 hours before administering the first dose of enteral L-citrulline. The second sample was collected 10 to 30 minutes before the last (12th) dose of enteral L-citrulline. These sampling times were designed to assess baseline and steady-state trough L-citrulline plasma concentrations.

For each infant, we also collected two urine samples via cotton balls placed in their diapers. The first urine sample was collected the day before administering the first enteral dose of L-citrulline. The second sample was collected between 4 and 8 hours following the last (12th) dose of enteral L-citrulline. The urine collection times were designed to assess baseline levels of nitric oxide metabolites (nitrite/nitrate) in the urine and whether levels of nitric oxide metabolites increased in response to 72 hours of L-citrulline enteral dosing.

### Sample processing and analyses.

All blood samples were centrifuged at 1500 g for 5 minutes at 4° C. The plasma was separated and stored at −80° C for later determination of concentrations of the amino acids, L-citrulline (μmol/L) and L-arginine (μmol/L). All amino acid analyses were performed by ARUP Laboratories (Salt Lake City, UT) via Liquid Chromatograph-Tandem Mass Spectrometry (LC-MS/MS) using the Sciex aTRAQ^™^ labeling method and an LC-MS/MS system comprising a Sciex API 4000 triple quadrupole mass spectrometer and a Shimadzu High-Performance Liquid Chromatography System. Amino acids were separated with a C18 chromatographic column and identified using their characteristic retention times and mass transitions. To allow accurate quantification, each analytical run included a calibration curve for each reported amino acid, and internal standards were used to correct for ionization differences and ion suppression.

Each urine sample was aliquoted into two separate tubes (minimum 0.5 ml urine per tube), then stored at −80° C. One of the aliquoted urine samples was used to determine urine creatinine (Cr). Urine Cr analysis was performed by either ARUP Laboratories (Salt Lake City, UT) or the Central Laboratory at the University of Utah Health Hospital (Salt Lake City, UT).

The second aliquoted urine sample was used to determine urine nitric oxide metabolites, nitrites/nitrates (NOx). For urine NOx analysis, using methods as previously described^18^, urine samples were diluted 1:10 with sterile water, then injected (20 μL) into the reaction chamber of a chemiluminescent NO analyzer (Seivers). The reaction chamber contained vanadium (III) chloride in 1 M HCl heated to 90° C to reduce nitrite and nitrate to NO gas. N_2_ gas was used to transfer the NO gas to a gas bubble trap where HCl vapor was removed by 1 M NaOH. A standard curve was generated for each analytical run by adding known amounts of NaNO_3_ to distilled water and assaying as described for the urine samples. Urine NOx was corrected for urine Cr (urine NOx/urine Cr).

### Pharmacokinetic analysis.

Dosing regimen (using median dose) for the current study was used to obtain simulated concentration-time data and corresponding profiles of L-citrulline using R (version 4.1.0) via package “linpk”. Parameter values for clearance, volume of distribution and absorption rate constant were obtained from a previous study and were fixed at 0.6L/h, 0.9L and 0.95, respectively^17^. The concentration time data generated from the simulations were overlaid and visually compared to plasma L-citrulline steady-state trough concentrations obtained from patients in this study.

### Statistical analyses.

Wilcoxon’s signed rank test was used to compare plasma concentrations of L-citrulline and arginine obtained at baseline to plasma concentrations obtained prior to the 12th L-citrulline dose and to compare values of urine NOx/urine Cr obtained at baseline to those obtained after L-citrulline treatment. A p-value of < 0.05 was considered significant.

## Results

### Subject characteristics.

A total of 6 premature infants were enrolled in this study. Slow enrollment and budgetary limitations, which precluded updating the Certificate of Analysis on the L-citrulline product used in this study, necessitated the termination of the study after the sixth study patient was enrolled. The median birth weight was 1050 grams (range 769–1430 g) and the weight at the time of study was 1487.5 grams (range 1330–1880 g) (Table 1). All six patients were receiving human milk feedings, median volume of 142 ml/kg/d (range 132–150 ml/kg/d), and none were receiving parenteral nutrition.

### Plasma amino acid and urine NOx data.

**Figure 1:**
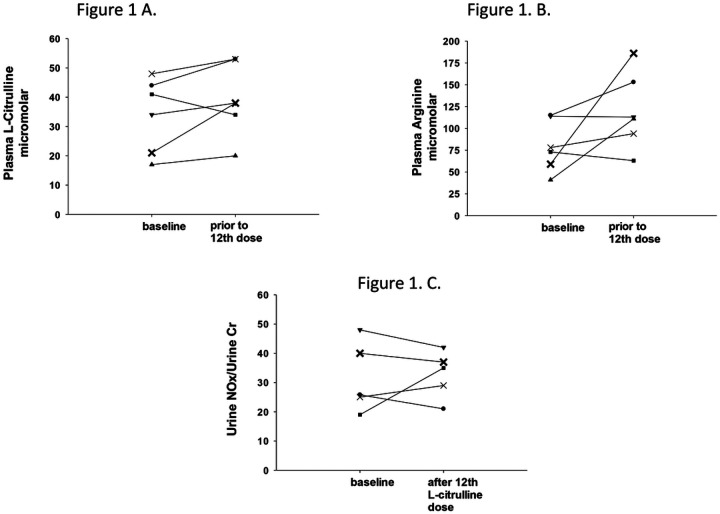
A. Plasma L-citrulline concentrations (mmol/L) of individual subjects collected at baseline and prior to the 12^th^ dose of L-citrulline. B. Plasma arginine concentrations (mmol/L) of individual subjects collected at baseline and prior to the 12^th^ dose of L-citrulline. C. Urine NOx/Urine Cr levels of individual subjects collected before (baseline) and after the 12^th^ dose of L-citrulline.

**Figure 2:**
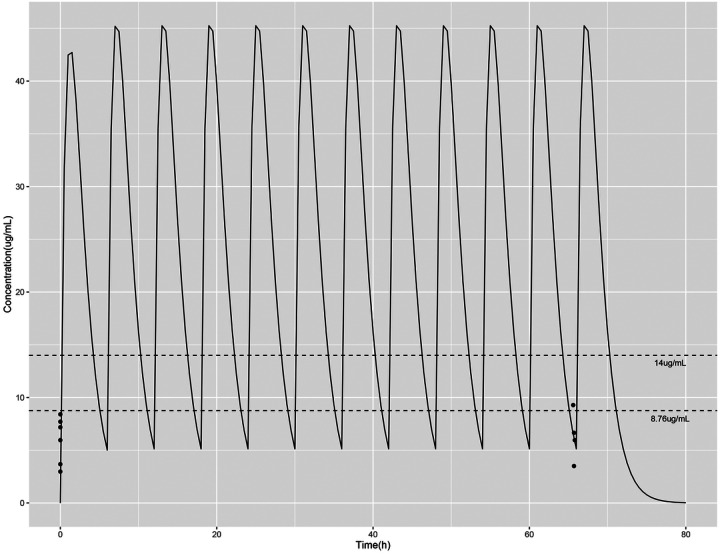
Observed/predicted plasma L-citrulline concentration (mg/mL) time profiles. Solid black lines represent predicted plasma L-citrulline concentrations. Dotted black lines represent 8.76 mg/ml (50 mmol/L) and 14 mg/mL (80 mmol/L) concentrations. Black dots are the observed L-citrulline concentrations (mg/mL) collected at study time points (i.e. at baseline and prior to the 12^th^ dose of L-citrulline). Note that 2 subjects had the same L-citrulline concentration of 9.3 mg/ml (53 mmol/L) at the second study time point so that dot reflects the data for 2 subjects.

Basal plasma concentrations were obtained between 30 minutes to 5 ½ hours prior to administering the first dose of L-citrulline, and a second plasma concentration was obtained between 18–27 minutes prior to the last, 12^th^, dose of L-citrulline. There was no difference between the baseline L-citrulline plasma concentrations (median 37.5 mmol/L, IQR 20–45 mmol/L), and the plasma concentrations obtained prior to the last dose of L-citrulline (median 38 mmol/L, IQR 30.5–53 mmol/L, p=0.17) for the combined group of six patients. There was also no difference between baseline plasma arginine concentrations (median 75.5 mmol/L, IQR 54.5–114.3 mmol/L), and plasma arginine concentrations obtained prior to the last dose of L-citrulline (median 112 mmol/L, IQR 86–161.3 mmol/L p=0.12) in this study cohort. When considering each individual patient as shown in Figure 1 A, for five of the six subjects the plasma L-citrulline concentration obtained before the last dose of L-citrulline treatment was higher than that of the subject’s baseline plasma L-citrulline concentration. The increase from baseline was variable, ranging from an increase of 10% to 81%. As shown in Figure 1 B, for four of the subjects, the plasma arginine concentration obtained prior to the last dose of L-citrulline treatment was higher than the plasma arginine concentration obtained at baseline for that individual subject. The percent increase from baseline plasma arginine concentration was variable, ranging from an increase of 21% to 215%. Figure 2 shows good agreement between the plasma L-citrulline concentration-time profile simulated in the single-dose study and those observed from the subjects in this multi-dose study. L-citrulline plasma concentrations were excluded for one subject because a protocol deviation occurred that resulted in the second L-citrulline plasma concentration being collected at a timepoint offset by 3 hours from the other five subjects.

Five of the six premature infants enrolled in this study provided two urine NOx/Cr levels. The first urine sample from these five subjects was collected before administering the first dose of L-citrulline or shortly after the first dose of L-citrulline was given. The first urine sample collected from one patient was lost. All six premature infants enrolled in this study provided urine NOx/Cr levels from urine collected between 6 ¼ and 7 ½ hours after administering the last study dose of L-citrulline. There was no difference between the urine NOx/Cr levels (median 25.8, IQR 22–44) obtained as a baseline and the urine NOx/Cr levels obtained after the last dose of L-citrulline (median 36, IQR 27–46.6) for the combined group of 5 subjects that provided two samples (p=0.89). When considering values for each of the individual subjects, as shown in Figure 1 C, only 2 subjects, had a NOx/Cr value obtained after the last dose of L-citrulline treatment that was higher than that subject’s baseline urine NOx/Cr value.

### Physiologic parameters, tolerability and safety data.

Table 2 summarizes respiratory support at the start and end of the 72 hours of L-citrulline treatment for all six subjects. Respiratory support changed slightly, if at all, between the start and end of the study for each of the subjects (Table 2).

All six subjects tolerated feedings throughout the study. None of the subjects developed vomiting, diarrhea, or evidence of necrotizing enterocolitis or gastrointestinal bleeding. No subject was evaluated for sepsis during the study drug treatment.

**Figure 3:**
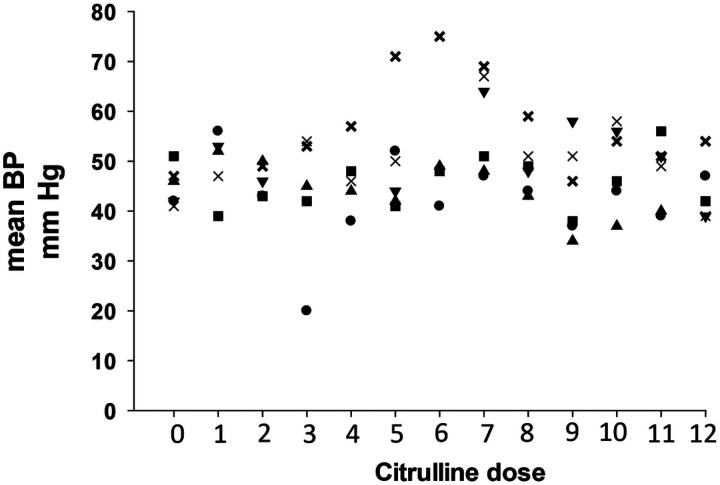
The mean BP measurements (mm Hg) of individual subjects recorded at baseline (dose 0) and with each of the 12 doses of L-citrulline administration.

Figure 3 illustrates the mean BP measurements obtained as a baseline during the 12 hours before administering the first L-citrulline and throughout the 72 hours of drug administration. Each BP measurement was coordinated with each q 6-hour dose of L-citrulline. Specifically, the BP measurements were obtained during the time of 30 minutes before to 50 minutes after each of the 12 doses of L-citrulline. Only one subject experienced a decrease in mean BP to a level more than 25 mmHg below that subject’s baseline. The low BP recording was obtained 30 minutes after the 3^rd^ dose of L-citrulline. The assessment by the caregivers at the time of the lower BP recording was that the subject was asymptomatic and that no treatment was required. That subject’s BP increased, returned to and remained at acceptable levels for the remainder of the study. The adverse event was reported to the DSMB as a non-serious, mild adverse event, possibly related to the study drug administration. No other adverse events occurred during the study.

## Discussion

Premature infants with BPD-PH are an understudied group of patients desperately needing new therapies. The current use of PH-targeted medications in neonates with BPD-PH is off-label. RCTs are needed to evaluate the safety and effi cacy of PH-targeted pharmacotherapies in premature infants with or at risk of developing BPD-PH. Prior to performing RCTs, PK studies need to be conducted to guide the choice of doses to be evaluated for safety and effi cacy^13^. As a first step in developing oral L-citrulline as a potential therapy for BPD-PH, we recently completed a study that simulated the PK profile of a multi-dosing regimen from data from a single dose of enterally administered L-citrulline in premature infants at risk of developing BPD-PH^17^. The current study showed that plasma L-citrulline concentrations observed from even doses given four times per day were in agreement with those predicted from the simulation in our previous single-dose study^17^. Although individual concentrations for only two subjects (33%) in this study with multiple doses achieved our target of 50–80 μmol/L (8.76–14 μg/ml), it can be seen from simulated concentration-time profiles that all subjects most likely reached the target concentrations, but not at the time that the sample was acquired. Samples collected later in an individual dosing interval may be better choices for therapeutic drug monitoring to assess if patients attain selected concentration targets.

Several previously published findings influenced our decision to target steady-state L-citrulline plasma concentrations of 50–80 μmol/L in this study. We were aware that investigators interested in developing L-citrulline as a treatment to prevent post-operative PH in infants and children with congenital heart disease undergoing cardiopulmonary bypass had identified plasma L-citrulline concentrations above 37 μmol/L as being protective^19, 20^. A recent study also found that premature infants with BPD-PH had median plasma L-citrulline concentrations of 21 μmol/L, whereas median plasma L-citrulline concentrations were higher, 36 μmol/L, in infants with BPD who did not develop PH^21^. Findings from these studies suggest that achieving plasma L-citrulline concentrations above 37 μmol/L might be needed to prevent various forms of PH in children. Moreover, oral treatment with L-citrulline inhibited PH development in chronically hypoxic piglets who achieved 50–100% increases from their basal plasma L-citrulline concentrations^15, 16^. This latter finding led to our choice to target steady-state L-citrulline plasma concentrations at least 50–100% above basal levels of our patient population, using the median basal plasma L-citrulline concentrations of patients in our previous study, 31 μmol/L. Notably, the basal L-citrulline plasma concentrations of the 6 patients in this study, median 37.5 μmol/L, were similar to those in our previous study and comparable to previously published basal levels in pediatric patients^22^.

The dosing strategy for this study was based on an optimal design dosage simulation-based methodology from our single-dose pharmacokinetic study^17, 23^. Simulations in our previous study predicted that enterally administering a L-citrulline daily dose of 150 mg/kg/d given once daily to eight times a day would achieve steady-state target trough plasma L-citrulline concentrations of 50–80 μmol/L (8.76–14 μg/ml) in premature infants at 32 ± 1 week postmenstrual age. In order to achieve plasma L-citrulline concentrations in the range of 80 μmol/L (14 μg/ml), doses of approximately 240 mg/kg/d are predicted to be needed. We considered practical issues when deciding the dosing frequency. The volume of each dose needed to be small enough to minimize the potential for emesis. In addition, since the long-range intent is that the L-citrulline will be administered for weeks to months, the frequency of dosing needs to not be overly burdensome for the caregiver. Therefore, a total daily dose of 240 mg/kg/d (total daily volume of 4.8 ml/kg/d) was divided into 60 mg/kg doses q 6 hours (1.2 ml/kg/dose given 4 × a day) which considered both the desired target L-citrulline plasma concentrations and practical considerations of drug administration.

Of note, L-citrulline is endogenously produced from amino acids, including glutamine and proline, by enterocytes in the proximal intestines^24^. It is possible that differences in total daily amounts of dietary protein administered and absorbed by the gastro-intestinal tract might have contributed to the range in patient plasma L-citrulline concentrations measured both at baseline and after 72 hours of L-citrulline administration. L-citrulline is considered a non-essential amino acid, so L-citrulline is not present in either infant formulas or parenteral nutrition. In addition, the normal human adult diet contains almost no L-citrulline, so the L-citrulline content in human milk is negligible^25^. Thus, other than the L-citrulline administered every 6 hours as part of this study, no patient should have received an exogenous source of L-citrulline that would have influenced their L-citrulline plasma concentrations. However, it should be considered that variability in the bioavailability of the enterally administered L-citrulline, i.e., variability between patients in the amount of L-citrulline absorbed by the gastro-intestinal tract, may have contributed to the variability in degree of increase from baseline L-citrulline plasma concentrations and help explain why the trough increased 50–100% above baseline in only one of the six patients (17%).

A sizable amount of circulating L-citrulline is converted in the kidney to L-arginine. Therefore, it is unsurprising that for most of the patients in the study the change in plasma concentrations of L-arginine paralleled those for L-citrulline. However, unlike L-citrulline, L-arginine is a semi-essential amino acid that must be provided as part of the human diet^26, 27^. This is particularly true for premature infants with a limited ability to produce L-arginine endogenously and reliance on exogenous, i.e. dietary sources, to maintain plasma L-arginine levels that are considered adequate for their needs^27^. Since the volume of human milk feedings ranged from 132–150 ml/kg/d, amounts of exogenously supplied dietary L-arginine might have contributed to the variability in plasma L-arginine concentrations between subjects in this study.

Oral administration of L-citrulline has been shown to increase the rate of plasma NO production in both children and adults^28, 29^. Urinary nitrites and nitrates have been used to reflect systemic NO production^30, 31^. Therefore, it may seem surprising that only two patients in this study had a change in urine NOx/Cr that paralleled increases in plasma concentrations of L-arginine and L-citrulline. Notably, other investigators have been unable to detect significant changes in urine nitrites and nitrates without administering very high doses of oral L-citrulline to adults (3 g L-citrulline twice a day for a week)^32^. Moreover, although rates of NO production increased with L-citrulline administration, plasma NOx concentrations did not change^28^. Both plasma and urine nitrite and nitrate concentrations are known to be confounded by dietary sources, limiting the ability of these measurements to reflect systemic or pulmonary NO production. Consequently, urine NOx/Cr measurements should not be relied on to accurately reflect changes in systemic or pulmonary NO production. Thus, even though we did not find an increase in Urine NOx/Cr with L-citrulline treatment in our group of 6 patients, it should not be concluded that systemic or pulmonary NO production did not increase with L-citrulline treatment in any or all the subjects.

Importantly, this study showed that this fragile patient population well tolerated the L-citrulline dosage strategy used in this study. For example, none of the patients developed evidence of gastrointestinal intolerance with the L-citrulline dosage strategy. Nor did any of the patients experience a decrease in systemic BP that warranted any therapeutic intervention. In addition, with exception of one subject who had a slight increase in respiratory support, the respiratory support was unchanged or reduced during study drug administration. This latter finding is important because neonates with respiratory diseases are at risk of developing pulmonary edema when total daily fluid volumes are increased. Maintaining respiratory stability in our patients in the face of the additional fluid volume required to administer the L-citrulline, 4.8 ml/kg/d in this case, is an important consideration.

We intended to study a population of premature infants at risk of developing BPD-PH. Premature infants born at ≤ 28 weeks gestation are known to be at greater risk of developing BPD than those born at more mature gestational ages^33, 34^. Current definitions of BPD are based on the respiratory support needs at 36 weeks postmenstrual age, with the definition of no BPD being the lack of respiratory support at 36 weeks of postmenstrual age^33, 34^. Therefore, infants were eligible if they had been born at ≤ 28 weeks gestation, and required respiratory support on the day of and for 14 days prior to study initiation. We chose to study infants at 32 ± 1 weeks postmenstrual age and not wait until 36 weeks, the postmenstrual age at which BPD is diagnosed. This choice is because of our ultimate goal to perform an RCT to determine whether starting oral L-citrulline treatment at or before 32 weeks postmenstrual age will reduce the percentage of neonates with BPD who have evidence of PH when they are ≥ 36 weeks postmenstrual age.

This study has limitations. One limitation is the small number of patients that we could enroll. Future Phase II studies need to be conducted in a larger number of patients to more completely evaluate the dosing strategies needed to achieve targeted steady-state plasma concentrations. Consistent with the experience of other investigators^35^, we believe that one obstacle to successfully enrolling fragile patients into phase I studies like ours is that their parents were reluctant to grant consent for a study that had no direct benefit. Another limitation is that because we performed the study with premature infants, we used a limited sampling strategy and collected only two blood samples per patient. We were also limited by not knowing the actual steady-state L-citrulline concentrations that will achieve therapeutic effi cacy. Nor did we include efficacy end points. Indeed, it should be emphasized that no phase III RCT has been performed to evaluate and prove that targeting the NO pathway with any medication, including L-citrulline, is an effi cacious therapy for infants with BPD-PH. This phase I study aimed to provide information to better inform the design of future phase II and phase III RCTs.

In summary, this study builds on the data from our previous single-dose PK study conducted in premature infants at risk of developing BPD-PH^17^. These data provide evidence that simulations based on single-dose pharmacokinetics can adequately predict concentrations for multiple dosing strategies. Moreover, these data can inform the choice of plasma sampling times and doses needed to achieve target L-citrulline plasma concentrations. Ultimately, phase III RCTs must be conducted to evaluate whether using an oral L-citrulline dosing strategy that achieves steady-state plasma L-citrulline concentrations of 50–80 μmol/L effectively prevents or ameliorates BPD-PH. Prior to performing a phase III RCT, a phase II RCT should be performed to evaluate the safety and provide some pharmacodynamic and preliminary effi cacy information. Taken together, the results of this and our previous study provide the critical information needed to design a phase II RCT and take the next step towards evaluating the use of oral L-citrulline as a potential treatment to inhibit BPD-PH in premature infants.

## Figures and Tables

**Table 1.** Patient Characteristics

Postmenstrual age at study date	32 0/7 (31 1/7, 33 0/7)
Birth weight (grams)	1050 (769, 1430)
Weight at study date (grams)	1487.5 (1330, 1890)
Sex	
Female	2 (33%)
Male	4 (67%)
Race	
Not Hispanic or Latino	4 (67%)
Other	0
Asian	0
Black	0
Unknown	2 (33%)
Ethnicity	
Caucasian	4 (67%)
Hispanic or Latino	0
Not Hispanic or Latino	0
Not Reported	2 (33%)
Type of feeding	
Human milk	6 (100%)
Premature infant formula	0
Volume of feedings (ml/kg/d)	142 (132, 150)

Values presented are counts (%) for categorical variables or median (min,max) for continuous variables

**Table 2.** Respiratory support for each patient at the start and end of the 72 hour period of L-citrulline dosing.

Patient	1		2		3		4		5		6	
L-citrulline dosing	Start	End	Start	End	Start	End	Start	End	Start	End	Start	End
Respiratory Support Mode	CPAP	CPAP	CPAP	CPAP	CPAP	CPAP	HFNC	HFNC	CPAP	RA	HFNC	HFNC
MAP (cm H_2_O) or Flow (L/min)	11	10	12	12	10	10	5	4	5	0	5	6
FiO_2_	24%	24%	23%	23%	29%	30%	24%	28%	21%	21%	24%	27%

CPAP = Continuous positive airway pressure

HFNC = High flow nasal cannula

RA = room air (i.e., no respiratory support)

FiO_2_ = fractional inspired oxygen
